# Association between malnutrition and contrast-associated acute kidney injury in congestive heart failure patients following coronary angiography

**DOI:** 10.3389/fnut.2022.937237

**Published:** 2022-11-17

**Authors:** Ming Ying, Junqing Yang, Zhidong Huang, Yihang Ling, Bo Wang, Haozhang Huang, Qiang Li, Jin Liu, Yong Liu, Zhujun Chen

**Affiliations:** ^1^Department of Cardiology, Guangdong Cardiovascular Institute, Guangdong Provincial People’s Hospital, Guangdong Academy of Medical Sciences, Guangzhou, China; ^2^Department of Guangdong Provincial Key Laboratory of Coronary Heart Disease Prevention, Guangdong Cardiovascular Institute, Guangdong Provincial People’s Hospital, Guangdong Academy of Medical Sciences, Guangzhou, China; ^3^The Second School of Clinical Medicine, Southern Medical University, Guangzhou, China

**Keywords:** malnutrition, congestive heart failure, contrast-associated acute kidney injury, mortality, coronary angiography (CAG)

## Abstract

**Background:**

Previous studies have shown that malnutrition is very common in patients with congestive heart failure (CHF) and is closely related to the occurrence of acute kidney injury. However, the relationship between malnutrition and contrast-associated acute kidney injury (CA-AKI) is unclear.

**Method and results:**

We obtained data from 842 patients who were diagnosed with CHF following coronary angiography (CAG) or percutaneous coronary angiography (PCI) and had follow-up information from January 2013 to February 2016. The patients were divided into 3 groups according to the Controlling Nutritional Status Score before CAG or PCI procedure (Group 1: Normal; Group 2: Mild Malnutrition; Group 3: Moderate to Severe Malnutrition). The main endpoint was CA-AKI. Univariate and multivariable logistic regression analyses were performed. 556 (60.0%) patients suffered from malnutrition before CAG or PCI. During a median follow-up of 2.1 years, A total of 49 (5.82%) patients developed CA-AKI. Additionally, 5 (1.75%), 26 (6.27%) and 18 (12.77%) events were documented in patients with normal, mild and moderate or severe malnutrition, respectively (*p* < 0.01). In multivariable-adjusted models, patients with malnutrition showed a significantly higher incidence of CA-AKI than those in the normal group.

**Conclusion:**

Malnutrition is an independent risk factor for CA-AKI in CHF patients following CAG.

## Introduction

Contrast-associated acute kidney injury (CA-AKI), representing one third of all hospital-acquired acute kidney injuries, is associated with poor short-term and long-term outcomes (1–3). Adequate hydration is the most common way to prevent CA-AKI in patients following coronary angiography (CAG), while over hydration might induce acute heart failure (AHF) in patients with congestive heart failure (CHF) (4). Therefore, early identification of high-risk groups and active intervention are essential to preventing CA-AKI in chronic heart failure patients following CAG. Malnutrition, defined as any nutritional imbalance, is a major complication in hospitalized patients and is associated with increased healthcare costs and mortality, especially among CHF patients (5). The Controlling Nutritional Status Score (CONUT) was developed by Ulibarri et al. in 2005 as a screening tool for the nutritional status of hospitalized patients (6). Previous studies have shown that malnutrition is closely related to the occurrence of acute kidney injury (7–9). However, there has been a lack of research of the relationship between malnutrition and CA-AKI in congestive heart failure patients following CAG.

## Materials and methods

### Study population

**FIGURE 1:**
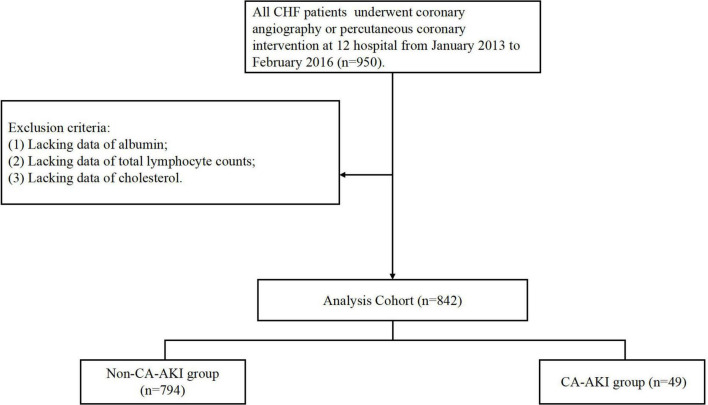
Flow chart.

The present study was a prospective, multicenter, observational cohort study of patients who were admitted to one of twelve teaching hospitals in the Guangdong, Fujian and Xinjiang Provinces of China from January 2013 to February 2016 (NCT01402232; Supplementary Table 1). We included total of 950 patients who underwent coronary angiography (CAG) or percutaneous coronary angiography (PCI) and were diagnosed with CHF. A total of 842 patients were enrolled in the final analysis after excluding patients combined with conditions as follow: (a) lacking data of albumin (*n* = 67); (b) lacking data of total lymphocyte counts (*n* = 9); (c) lacking data of cholesterol (*n* = 32; Figure 1). The study and all of its protocols were approved by the institutional Ethics Research Committee of Guangdong Provincial People’s Hospital (No. GDREC2012141H). All patients gave written informed consent before undergoing CAG.

### Malnutrition screening tools

The CONUT score that was developed by Ulibarri et al. in 2005 as a screening tool for the nutritional status of hospitalized patients, and has been validated for a wide range of populations, was applied to assess the nutritional state of patients with CHF (10, 11). It automatically assesses nutritional status, taking into account serum albumin, cholesterol, and total lymphocyte count (Supplementary Table 2). A score of 0–1 indicates a normal nutrition state, 2–4 reflects mild malnutrition, and 5–12 indicates moderate to severe malnutrition (12).

### Data collection and definitions

The assessment of malnutrition was carried out before CAG or PCI according to the CONUT score. CAG procedures were performed according to standard clinical practice guidelines using standard guide catheters, guide wires, balloon catheters, and stents via the femoral or radial approach (4). The intravenous contrast agent administered was at the discretion of the interventional cardiologist. All patients received non-ionic, low-osmolarity contrast agents. The treatment and hydration protocols were based on current guidelines at the discretion of the cardiologists according to clinical conditions. High-risk patients were recommended to receive a continuous adequate intravenous infusion of isotonic saline or sodium bicarbonate solution at a rate of 1 ml/kg per hour (0.5 ml/kg per hour if the left ventricular ejection fraction was <35% or the New York Heart Association score was >2) for 12 h before and 12–24 h after the procedure (4). Preprocedural oral hydration volumes were recorded.

Baseline levels of blood indicators were measured. Serum creatinine was measured before CAG, on days 1, 2, and 3 postoperatively, and upon hospital discharge. The start time and end time of the procedure and the urine output during the 24 h after the procedure were recorded. Preprocedural interventions (i.e., normal saline injections and furosemide or statin administration), PCI technique, contrast agent details (non-ionic, low-osmolality, or isotonic), and contrast dose were selected at the discretion of the attending cardiologist based on their routine procedures and according to current practice guidelines. Patients were excluded if they received an intravenous contrast agent within 72 h after the procedure.

After discharge, adverse clinical events were assessed and registered by the attending physician or trained research assistant for a follow-up period of over 12 months. Data on patient and procedural characteristics was also obtained through original records and hospital electronic medical records. We calculated the estimated glomerular filtration rate (eGFR) by applying the Modification of Diet in Renal Disease (MDRD) equation (13). CHF was defined as a New York Heart Association (NYHA) class > 2 or Killip class > 1 (14).

### Clinical outcomes

The primary endpoint was CA-AKI, defined as an increase in the level of Scr >0.3 mg/dl or >50% from the baseline level within the first 48 h after the procedure. The secondary endpoints were as follows: (1) 30-day mortality; (2) 1-year mortality; and (3) in-hospital dialysis.

### Statistical analysis

Descriptive statistics was applied, and the data were reported as the mean ± SD deviation in case of normal distribution and as the median and interquartile range when non-normally distributed. Categorical variables were expressed as numbers (percentages). The *t*-test and one-way ANOVA were used for comparisons of continuous data between the two groups, and comparisons of categorical variables between groups were performed by using a chi-square test. The associations between malnutrition and the study endpoints were assessed by univariate and multivariable logistic regression analysis, with model adjustments to include variables associated with known poor prognosis based on clinical plausibility. The results were presented as OR (95% CI). All *P* values <0.05 were considered significant. The decision curve analysis was adopted to quantify the predictive abilities of the multivariable logistic regression models (including multivariable logistic regression model and model added CONUT). The partial effect plot was used to express the value of CONUT in full model. The association between CA-AKI and the CONUT score level were evaluated on a continuous scale with restrictive cubic spline based on multivariable logistic regression model. The interaction between age and CONUT for prediction CA-AKI, also CRP and CONUT for prediction CA-AKI was evaluated with partial effect plot. The relative importance of item in CONUT were calculated, each item were included and removed, respectively, in full model and then Likelihood chi square, and Nagelkerke R2 value were calculated. All statistical analyses were performed using R (ver. 4.0.1).

## Results

### Baseline characteristics

Among the 842 patients, included in the study, 556 (60.0%) suffered from malnutrition. The overall median age was 64.9 ± 11.3 years. Malnourished patients were older and more likely to be male. The baseline hemoglobin, total cholesterol, total lymphocyte count and albumin level were significantly lower in the group with malnutrition. We found that malnourished patients had worse underlying kidney function. More data on the baseline demographic and clinical characteristics of the patients are presented in Table 1.

**TABLE 1:** Baseline characteristics in patients stratified by nutrition station.

Characteristics	Normal nutrition	Mild malnutrition	Moderate to severe malnutrition	*P-value*
			
	*N* = 286	*N* = 415	*N* = 141	
Age, years	61.84 (11.27)	65.33 (10.95)	69.90 (10.38)	<0.001
Male, *n* (%)	199 (69.58)	320 (77.11)	102 (72.34)	0.077
Diabetes mellitus, *n* (%)	87 (30.42)	117 (28.19)	55 (39.01)	0.055
Hypertension, *n* (%)	158 (55.24)	239 (57.59)	87 (61.70)	0.446
Smoke, *n* (%)	99 (34.62)	152 (36.63)	59 (41.84)	0.344
SBP, mmHg	133.23 (22.31)	132.30 (22.70)	125.89 (24.40)	0.005
DBP, mmHg	78.21 (13.32)	76.53 (13.15)	73.68 (13.15)	0.004
BMI, kg/m^2^	24.05 (3.30)	23.41 (3.45)	22.50 (3.41)	0.001
Hemoglobin, g/L	137.73 (14.84)	132.13 (16.92)	119.99 (20.38)	<0.001
Total cholesterol, mmol/L	5.28 (1.19)	4.37 (1.15)	3.87 (1.09)	<0.001
Total lymphocyte count, 10^9^/L	2.23 (0.86)	1.70 (0.90)	1.20 (0.59)	<0.001
Albumin, mmol/L	39.59 (3.44)	35.72 (3.96)	29.88 (4.03)	<0.001
Serum creatine, medium (IQR)	89.02 (30.84)	74.73 (27.66)	64.58 (37.11)	<0.001
eGFR, ml/min/1.73 m^2^	81.77 (25.54)	76.23 (27.47)	62.23 (49.39)	<0.001
Uric acid, umol/L	395.99 (124.52)	400.60 (132.14)	412.36 (161.94)	0.577
C-reactive protein, mg/L	8.30 (20.28)	15.15 (43.18)	43.09 (60.12)	<0.001
Contrst dose, ml	102.94 (55.76)	105.16 (60.58)	99.46 (53.30)	0.591
Pre-Statin, *n* (%)	167 (58.39)	247 (59.52)	77 (54.61)	0.593
Pre-CCB, *n* (%)	24 (8.39)	49 (11.81)	16 (11.35)	0.572
Pre-ACEI/ARB, *n* (%)	98 (34.27)	158 (38.07)	50 (35.46)	0.004
CA-AKI, *n* (%)	5 (1.75)	26 (6.27)	18 (12.77)	<0.001

### Clinical outcomes

During hospitalization, 49 (5.82%) patients developed CA-AKI. In total, 5 (1.75%), 26 (6.27%) and 18 (12.77%) events were documented in patients with normal, mild or severe malnutrition states, respectively (*p* < 0.01). The incidence of CA-AKI and 1-year mortality were significantly increased in the malnutrition group. There were no significant differences in the incidence of dialysis and short-term mortality among the groups (Table 2).

**TABLE 2:** CA-AKI incidence and clinical outcomes.

Events	Overall	Normal	Mild	Moderate or severe	*P-value*
				
	*n* = 842	*n* = 286	*n* = 415	*n* = 141	
CA-AKI, *n* (%)	49 (5.82)	5 (1.75)	26 (6.27)	18 (12.77)	<0.001
30-day death, *n* (%)	8 (1.05)	2 (0.75)	3 (0.82)	3 (2.27)	0.313
1-year death, *n* (%)	41 (5.37)	6 (2.25)	18 (4.93)	17 (12.98)	<0.001
In-hospital dialysis, *n* (%)	11 (1.39)	3 (1.11)	4 (1.04)	4 (2.88)	0.253

**FIGURE 2:**
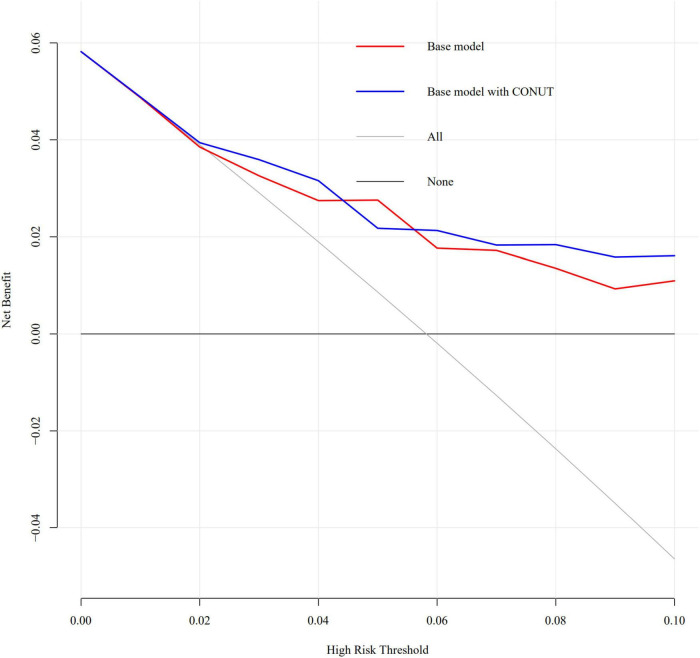
Decision curve analysis of full model and model without malnutrition.

**FIGURE 3:**
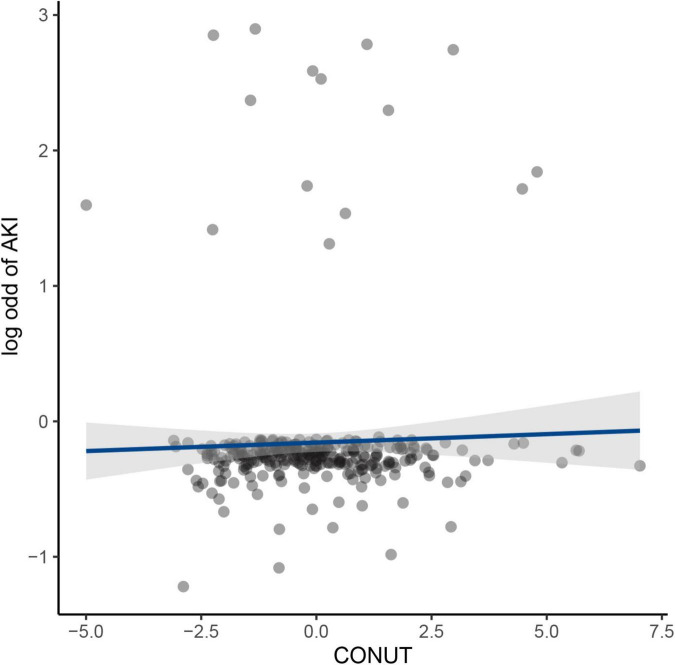
Partial effect plot in full model for Controlling Nutritional Status Score.

**FIGURE 4:**
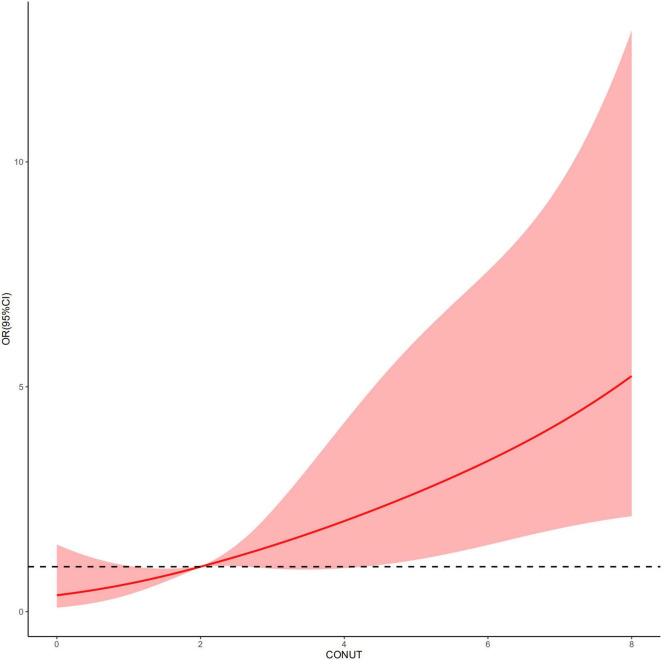
Restrictive cubic spline based on multivariable logistic regression model.

An increased CONUT value was associated with higher risk of CA-AKI. The univariate and multivariable logistic regression models showed that different levels of malnutrition were associated with the occurrence of CA-AKI in patients with CHF following CAG after adjusting for clinical variables (Table 3). In multivariable-adjusted model, patients with mild (2 ≤ CONUT score ≤ 4) [OR (95%CI) 3.15 (1.28-9.53)] and moderate (5 ≤ CONUT score ≤ 12) [OR (95%CI) 4.41 (1.57-14.45)] malnutrition showed a significantly higher incidence of CA-AKI than those in the normal group (*p* = 0.022 and 0.008, respectively). In decision curve analysis, model added CONUT showed higher net benefit when high risk threshold more than 5.5% (Figure 2). The partial effect plot showed the positive value of CONUT for higher predictive ability of CA-AKI (Figure 3). We observed the risk of CA-AKI increase with increase of the CONUT score level in the multivariable logistic regression model of restrictive cubic spline (Figure 4). We observed no significant evidence of interaction between CRP and CONUT (Supplementary Figure 1). We did not find any heterogeneity between age and CONUT for CA-AKI (Supplementary Figure 2). The importance of each item of the CONUT score were displayed (Supplementary Table 3).

**TABLE 3:** Univariate and multivariate logistic regression analysis of the association between malnutrition and CA-AKI.

Risk factors	Univariate		Multivariable	
		
	OR (95% CI)	*P*-value	OR (95% CI)	*P-value*
Age	1.04 (1.01–1.07)	0.006	1.02 (0.99–1.05)	0.154
Male	1.24 (0.65–2.60)	0.534		
BMI	0.98 (0.88–1.09)	0.990		
Diabetes mellitus	1.46 (0.79–2.62)	0.213	1.28 (0.67–2.37)	0.446
Smoke	1.70 (0.95–3.04)	0.072		
CKD	2.82 (1.44–6.05)	0.004		
IABP	4.24 (1.82–9.00)	<0.001	3.95 (1.65–8.74)	0.001
Hypertension	1.56 (0.86–2.96)	0.153		
Contrast dose	1.00 (1.00–1.01)	0.574	1.00 (1.00–1.01)	0.513
SBP	1.00 (0.99–1.01)	0.894		
DBP	1.00 (0.98–1.02)	0.742		
Hemoglobin	0.98 (0.96–1.00)	0.001	0.99 (0.98–1.01)	0.459
LYM,10^9^/L	0.80 (0.52–1.13)	0.258		
Urine acid	1.00 (1.00–1.00)	0.011		
Albumin	0.89 (0.84–0.95)	<0.001		
Total cholesterol	0.83 (0.64–1.06)	0.154		
Scr	1.01 (1.00–1.01)	<0.001	1.01 (1.00–1.01)	0.013
CRP	1.01 (1.00–1.02)	0.017	1.01 (1.00–1.02)	0.041
eGFR	0.98 (0.97–0.99)	0.001		
ACEI or ARB	0.32 (0.14–0.67)	0.004		
Mild malnutrition	1.36 (1.20–1.54)	<0.001	3.15 (1.28–9.53)	0.022
Moderate to severe malnutrition	3.76 (1.55–11.21)	0.007	4.41 (1.57–14.45)	0.008

Adjusting by age, diabetes mellitus, IABP, hemoglobin and serum creatine. BMI, body mass index; CHF, chronic heart failure; CKD, Chronic kidney disease; IABP, intra-aortic ballon pump; SBP, systolic blood pressure; DBP, diastolic blood pressure; LYM, lymphocyte count; SCR, serum creatine; CRP, c-reaction protein; eGFR, estimated glomerular filtration rate; ACEI, angiotensin-converting enzyme inhibitors; ARB, angiotensin receptor blockers; OR, odds ratio.

## Discussion

To the best of our knowledge, this is the first study that attempts to investigate the relationship between malnutrition and CA-AKI in CHF patients. Our study shows that malnutrition levels were associated with an increased risk of developing CA-AKI in CHF patients. After adjusting for confounding factors, malnutrition levels were still a significant factor for CA-AKI in CHF patients.

A prospective observational study conducted by Li et al. included 46,549 in-hospital patients, and found that malnutrition is widespread among hospitalized patients and has a strong association with acute kidney injury (AKI) (7). Yu et al. enrolled 3,185 acute coronary syndrome patients and found that the risk of malnutrition was a helpful tool in identifying patients at high risk for AKI and mortality (8). Süleyman C et al. found a similar conclusion by enrolled in 360 consecutive patients with coronary angiography performed because of chronic coronary artery disease. They found that malnutrion were independently associated with the presence of CI-AKI in elderly patients (15). A prospective and observational study that included a total of 114 CHF patients reported that a poor nutritional status, as assessed using the CONUT score, is significantly associated with poor outcomes (16). In order to found a better tool to assess the influence of malnutrition to mortality, Shirley et al. simple tools (CONUT Score, Geriatric Nutritional Risk Index, and Prognostic Nutritional Index) and learned that CONUT Score have the best predictive power of mortality in patients with CHF (17). Our results are in agreement with these studies. Similarly to the previous reports, our study focused on malnutrition, and revealed that malnutrition is an independent risk factor for kidney dysfunction. However, our study included 842 CHF patients, and the sample size was significantly larger than that of the other studies on CHF patients. Moreover, we are the first prospective study to explore the relationship between malnutrition and CA-AKI.

Malnutrition is currently commonly defined as an acute, subacute or chronic nutritional state by The European Society for Clinical Nutrition and Metabolism (18). In this state, with or without inflammatory activity, different degrees of overnutrition or undernutrition will lead to changes in body composition and impaired bodily functions. Previous studies have revealed that the incidence of malnutrition among the elderly population is 5–12%, with the rate of 16–78% for elderly inpatients in developing countries and 29–61% in developed countries. The incidence of malnutrition in patients with cardiac insufficiency is 42–58% (10, 19–22).

A retrospective observational study revealed that malnutrition is common among patients with acute coronary syndrome and is strongly associated with increased rates of mortality and cardiovascular events. For mortality risk prediction, each of the 3 malnutrition scores had significant incremental prognostic value for the GRACE risk score. The highest incremental value was seen for the Control Nutritional Status (CONUT) score (12) that includes albumin (ALB), total cholesterol (TC), and whole blood lymphocyte (LYM) count, and is used to assess protein storage, calorie consumption and immune defense in the body (6).

A retrospective study found a strong independent association between increased neutrophil-to-lymphocyte ratios (NLRs) after cardiovascular surgery and AKI occurring in the first 7 days after surgery (23). A meta-analysis showed that low ALB could independently predict the occurrence of AKI, and correction of hypoproteinemia could prevent the occurrence of AKI (24).

The mechanisms underlying the association between malnutrition levels and CA-AKI are unclear. One potential mechanism may involve the body’s inflammatory response. LYM count is often considered an inflammatory response and immune status marker. Previous study suggested that inflammatory cells in general, and lymphocytes in particular are involved in the initiation, proliferation and recovery stages of AKI and infiltrate damaged kidney tissues and produce inflammatory mediators such as cytokines and chemokines (25). Another possible mechanism may involve a protein-calorie imbalance that could cause renal hemodynamic changes, reduce renal blood flow, decrease the glomerular filtration rate, and decrease the ability of renal tubules to excrete acid (26, 27).

Our study also has several limitations. First, we did not track changes in renal function among discharged patients. Second, whether improvements in malnutrition states could prevent CA-AKI was not assessed. More randomized clinical treatment studies on malnutrition and CA-AKI are needed.

## Conclusion

In summary, malnutrition is an independent risk factor for CA-AKI in CHF patients following CAG. The higher the degree of malnutrition, the greater the risk of developing CA-AKI.

## Data availability statement

The original contributions presented in this study are included in the article/Supplementary material, further inquiries can be directed to the corresponding authors.

## Author contributions

MY and JY conceived and designed the study. ZH, YLn, BW, HH, QL, JL, YLu, and ZC collected the data and performed the analysis. MY, JY, and ZC were involved in the writing of the manuscript and is responsible for the integrity of the study. All authors have read and approved the final manuscript.

## Supplementary material

Supplementary Figure 1 Partial effect plot of interaction between CRP and Controlling Nutritional Status for CA-AKI.

Supplementary Figure 2 Partial effect plot of interaction between age and Controlling Nutritional Status for CA-AKI.
