# Supplementary material for: Association between malnutrition and contrast-associated acute kidney injury in congestive heart failure patients following coronary angiography
Source: Front Nutr. 2022 Nov 17;9:937237. doi: 10.3389/fnut.2022.937237 (PMC9713008; doi:10.3389/fnut.2022.937237)
Supplement: Supplementary file 1 [file Table_1.doc]

| **Supplemental table 1.Hospitals participating in this study** |
| --- |
| 1.Department of Cardiology, Guangdong Cardiovascular Institute, Guangdong Provincial People's Hospital |
| 2. Department of Cardiology, First Affiliated Hospital, Sun Yat-Sen University |
| 3. Department of Cardiology, Sun Yat-Sen Memorial Hospital of Sun Yat-Sen University |
| 4.Department of Cardiology, Nanfang Hospital of Southern Medical University |
| 5.Department of Cardiology, Guangzhou General Hospital of Guangzhou Military Command |
| 6. Guangdong Medical College |
| 7.Department of Cardiology, Dongguan Kanghua Hospital |
| 8.Department of Cardiology, Dongguan People's Hospital |
| 9.Department of Cardiology, Maoming People's Hospital |
| 10.Department of Cardiology, Futian People's Hospital |
| 11.Department of Cardiology, Longyan City First Hospital |
| 12.Department of Cardiology, First People's Hospital of Kashgar |
