# Supplementary material for: Association between malnutrition and contrast-associated acute kidney injury in congestive heart failure patients following coronary angiography
Source: Front Nutr. 2022 Nov 17;9:937237. doi: 10.3389/fnut.2022.937237 (PMC9713008; doi:10.3389/fnut.2022.937237)
Supplement: Supplementary file 2 [file Table_2.doc]

**Suppelmentary Table 2. CONUT Score**

| Level | Normal nutrition | Mild malnutrition | Moderate malnutrition | Severe malnutrition |
| --- | --- | --- | --- | --- |
| Albumin(g/L) | ≥35 | 30-34.9 | 25-29 | <25 |
| Point | 0 | 2 | 4 | 6 |
| Lymphocyte count(/mm^2^) | >1600 | 1200-1599 | 800-1199 | <800 |
| Point | 0 | 1 | 2 | 3 |
| Total cholesterol(mg/dL) | >180 | 140-180 | 100-139 | <100 |
| Point | 0 | 1 | 2 | 3 |
| Total score | 0-1 | 2-4 | 5-8 | 9-12 |
