# Supplementary material for: Association between malnutrition and contrast-associated acute kidney injury in congestive heart failure patients following coronary angiography
Source: Front Nutr. 2022 Nov 17;9:937237. doi: 10.3389/fnut.2022.937237 (PMC9713008; doi:10.3389/fnut.2022.937237)
Supplement: Supplementary file 3 [file Table_3.doc]

**Suppelmentary Table 3. Relative importance of each item in CONUT Score**

| Model | Likelihood Chi square | Nagelkerke R2 value |
| --- | --- | --- |
| Full model | 99.11 | 0.183 |
| Model without Total cholesterol | 99.57 | 0.178 |
| Model without Lymphocyte count | 98.87 | 0.185 |
| Model without Albumin | 100.66 | 0.168 |
