# Supplementary figures and images for: Association between malnutrition and contrast-associated acute kidney injury in congestive heart failure patients following coronary angiography
Source: Front Nutr. 2022 Nov 17;9:937237. doi: 10.3389/fnut.2022.937237 (PMC9713008; doi:10.3389/fnut.2022.937237)

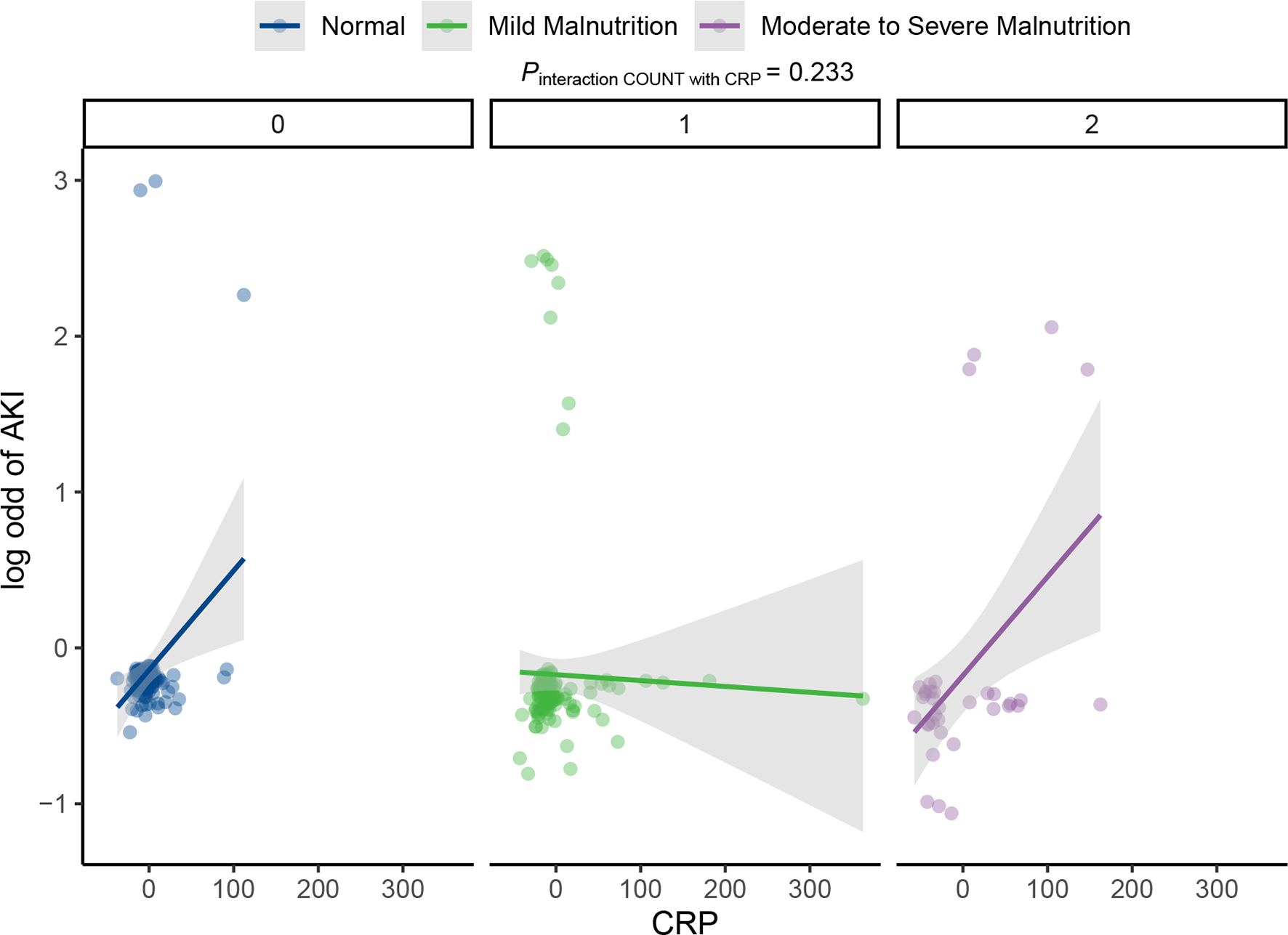

Supplement: Supplementary Figure 1 — Partial effect plot of interaction between CRP and Controlling Nutritional Status for CA-AKI. [file Image_1.TIF]

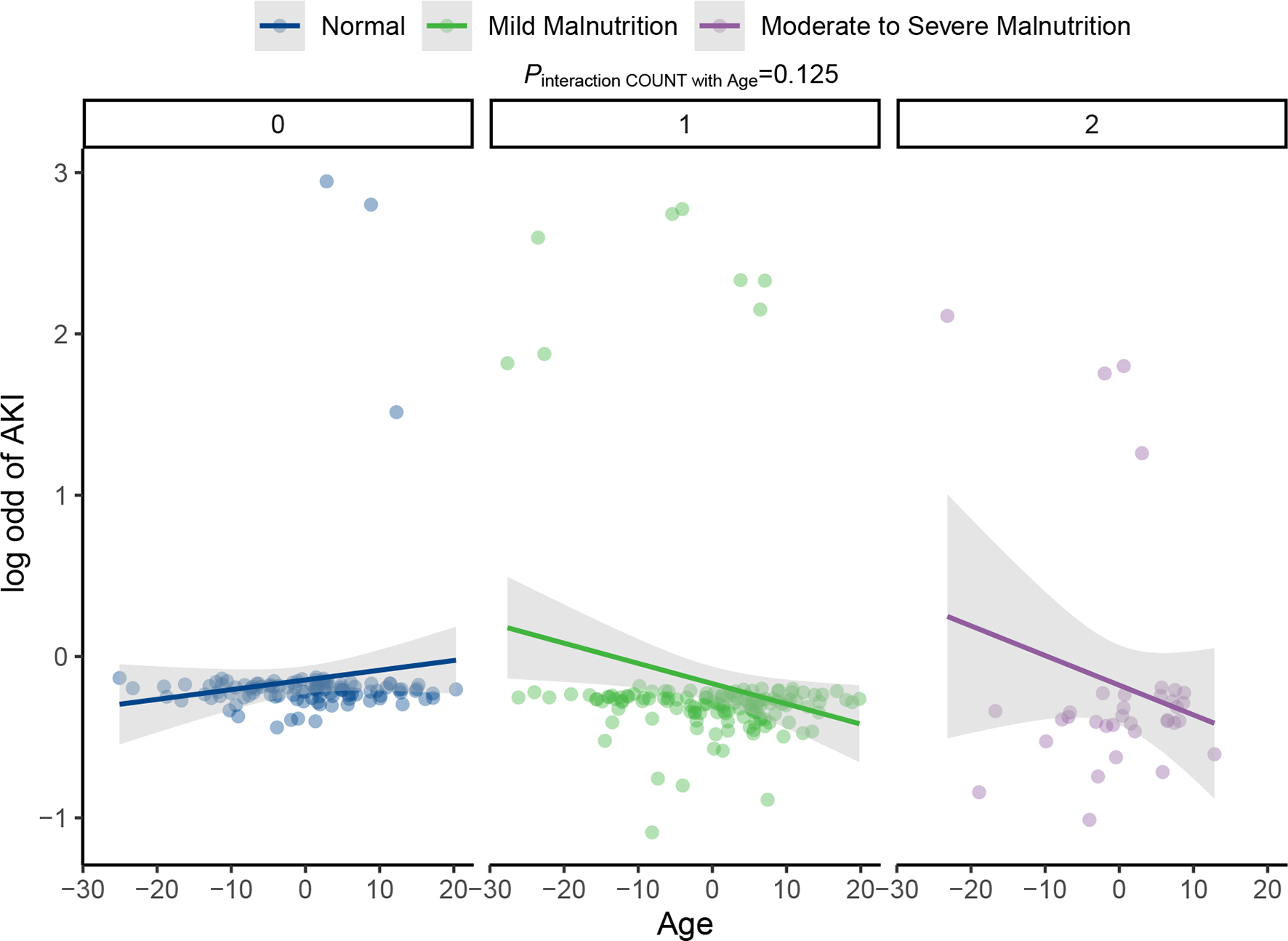

Supplement: Supplementary Figure 2 — Partial effect plot of interaction between age and Controlling Nutritional Status for CA-AKI. [file Image_2.TIF]
